# Comparative evaluation of salivary oxidative stress markers in oral leukoplakia and oral submucous fibrosis: A cross-sectional study

**DOI:** 10.6026/973206300223294

**Published:** 2026-06-30

**Authors:** Ashni Chatterjee, Sowjanya Gunukula, Mousami Kundu, Priya Sinha, Sameer Gupta, Gaurav Sharma

**Affiliations:** 1 Department of Oral Medicine and Radiology, Geetanjali Dental and Research Institute, Udaipur, Rajasthan, India; 2 Department of General Dentistry, Flomo Dental, Private Clinic, Independent Researcher, 295 Ukiah St, Lewisville TX 75056; 3 Department of Oral & Maxillofacial Surgery, Awadh Dental College and Hospital, Jamshedpur, Jharkhand, India; 4 Department of Oral Medicine and Radiology, Geetanjali Dental and Research Institute, Udaipur, Rajasthan, India; 5 Department of Oral & Maxillofacial Surgery, Inderprastha Dental College and Hospital, Sahibabad, Ghaziabad, Uttar Pradesh, India; 6 Department of Dentistry, SGT Dental College and Research Institute, Budhera, Gurgaon, Haryana, India

**Keywords:** Oral leukoplakia, oral submucous fibrosis (OSMF), oxidative stress, salivary biomarkers, malondialdehyde

## Abstract

Oral leukoplakia and oral submucous fibrosis (OSMF) are potentially malignant disorders with a significant risk of progressing to oral cancer. Oxidative stress is a key factor in their development, causing cellular damage and disease progression. Therefore, it is of interest to evaluate and compare salivary oxidative stress markers in patients with these conditions. Data shows high levels of oxidative stress and lower antioxidant levels in both conditions compared to healthy controls, with OSMF showing severe imbalance. Thus, we show salivary oxidative stress markers as potential non-invasive diagnostic tools for early detection and monitoring of oral potentially malignant disorders.

## Background:

Oral potentially malignant disorders (OPMDs) represent a group of lesions and conditions of the oral mucosa that carry an increased risk of malignant transformation into oral squamous cell carcinoma (OSCC). Among these, oral leukoplakia and oral submucous fibrosis (OSMF) are the most frequently encountered and clinically significant entities [1]. Oral leukoplakia is defined as a predominantly white lesion of the oral mucosa that cannot be characterized clinically or histopathologically as any other definable condition. It is considered the most common potentially malignant disorder, with varying rates of malignant transformation reported in the literature [2]. On the other hand, OSMF is a chronic, insidious disease characterized by progressive fibrosis of the oral mucosa, leading to stiffness, trismus and an increased risk of malignancy. Both conditions are strongly associated with deleterious oral habits such as tobacco use, areca nut chewing and alcohol consumption. The pathogenesis of these disorders is multifactorial and involves complex interactions between environmental carcinogens and host factors [3]. Among the various mechanisms implicated, oxidative stress has emerged as a crucial contributor in the initiation and progression of these lesions. Oxidative stress refers to an imbalance between the productions of reactive oxygen species (ROS) and the antioxidant defense mechanisms of the body. ROS, including free radicals such as superoxide anion, hydroxyl radicals and hydrogen peroxide, can cause significant damage to cellular components, includes lipids, proteins and DNA. This damage can lead to cellular dysfunction, mutagenesis and ultimately carcinogenesis [4]. In the context of oral leukoplakia and OSMF, chronic exposure to tobacco, areca nut and other irritants leads to excessive production of ROS. These reactive species can induce lipid peroxidation, protein oxidation and DNA strand breaks, thereby promoting epithelial dysplasia and fibrosis [5]. In OSMF, areca nut constituents such as arecoline stimulate fibroblast proliferation and collagen synthesis while simultaneously impairing collagen degradation, leading to excessive fibrosis. Oxidative stress further exacerbates this process by enhancing the cross-linking of collagen fibers and reducing tissue elasticity. Similarly, in oral leukoplakia, oxidative damage contributes to epithelial alterations, keratinization and dysplastic changes that may progress to malignancy. The human body is equipped with a robust antioxidant defense system to counteract oxidative stress. This system includes enzymatic antioxidants such as superoxide dismutase (SOD), catalase and glutathione peroxidase, as well as non-enzymatic antioxidants like uric acid, vitamin C and vitamin E [6]. These antioxidants neutralize ROS and protect cells from oxidative damage. However, in conditions like oral leukoplakia and OSMF, the balance between oxidants and antioxidants is often disrupted, resulting in increased oxidative stress and decreased antioxidant capacity. Therefore, evaluating oxidative stress markers and antioxidant levels can provide valuable insights into the pathophysiology and progression of these disorders [7]. Saliva has gained considerable attention as a diagnostic biofluid in recent years due to its non-invasive, easily accessible and cost-effective nature. It contains a wide array of biomarkers, including enzymes, proteins, electrolytes and oxidative stress markers, which reflect both local and systemic health status. Salivary analysis offers several advantages over serum-based investigations, such as ease of collection, minimal discomfort to patients and suitability for repeated sampling [8]. Importantly, saliva directly bathes the oral mucosa, making it particularly relevant for assessing oral diseases. Several studies have investigated salivary oxidative stress markers in oral potentially malignant disorders and OSCC. Markers such as malondialdehyde (MDA), a byproduct of lipid peroxidation, are commonly used to assess oxidative damage, while antioxidant enzymes like SOD and catalase are evaluated to determine the antioxidant defense status. Elevated levels of MDA and reduced levels of antioxidants have been reported in patients with oral leukoplakia and OSMF, indicating increased oxidative stress. However, the comparative evaluation of these markers between different OPMDs remains limited and there is a need for studies that directly compare the oxidative stress profiles of these conditions [9]. Understanding the differences in oxidative stress and antioxidant status between oral leukoplakia and OSMF may provide valuable information regarding their pathogenesis, severity and risk of malignant transformation. It may also aid in identifying potential biomarkers for early diagnosis, monitoring disease progression and evaluating therapeutic responses. Furthermore, such insights could contribute to the development of targeted antioxidant therapies aimed at reducing oxidative damage and preventing malignant transformation [10]. Despite the growing body of evidence supporting the role of oxidative stress in oral diseases, there remains a lack of comprehensive studies that simultaneously evaluate and compare salivary oxidative stress markers in oral leukoplakia and OSMF within the same population. Additionally, variations in study design, sample size and methodological approaches have led to inconsistent findings, highlighting the need for standardized and comparative research in this area. Therefore, it is of interest to determine the comparative levels of salivary oxidative stress markers and antioxidant status in patients with oral leukoplakia and oral submucous fibrosis and to assess their potential role as diagnostic and prognostic biomarkers in these potentially malignant disorders.

## Methodology:

## Study design and setting:

This cross-sectional comparative study was conducted in the Department of Oral Medicine and Radiology of a dental institution over a period of 6-12 months. The study aimed to evaluate and compare salivary oxidative stress markers among patients diagnosed with oral leukoplakia, oral submucous fibrosis (OSMF) and healthy controls.

## Sample size and study population:

A total of 100 participants were included in the study and divided into three groups as follows:

[1] Group I (Oral Leukoplakia): 35 patients clinically and histopathologically diagnosed with oral leukoplakia 

[2] Group II (OSMF): 35 patients clinically diagnosed with oral submucous fibrosis

[3] Group III (Control): 30 healthy individuals without any oral lesions or adverse habits

Participants were selected from patients attending the outpatient department. The sample size was determined based on previous studies evaluating oxidative stress markers, considering statistical significance and feasibility.

## Inclusion criteria:

[1] Patients aged between 18-60 years

[2] Patients clinically diagnosed with oral leukoplakia or OSMF

[3] Patients with a history of tobacco, areca nut, or related habits (for study groups)

[4] Healthy individuals without any oral lesions or systemic diseases (for control group)

[5] Individuals willing to provide informed consent

## Exclusion criteria:

[1] Patients with systemic diseases such as diabetes mellitus, cardiovascular disorders, or autoimmune diseases

[2] Patients on antioxidant supplements or medications affecting oxidative stress levels

[3] Patients with other oral potentially malignant disorders or malignancies

[4] Pregnant or lactating women

[5] Individuals with recent infections or inflammatory conditions

## Ethical considerations:

The study protocol was reviewed and approved by the Institutional Ethical Committee. Written informed consent was obtained from all participants prior to inclusion in the study. Confidentiality of patient data was strictly maintained throughout the study.

## Clinical examination and diagnosis:

A detailed case history was recorded for all participants, including demographic data, medical history and habit history (type, frequency and duration). Clinical examination was performed under adequate illumination using sterile instruments.

[1] Oral leukoplakia was diagnosed based on clinical features and confirmed by histopathological examination where required.

[2] OSMF was diagnosed clinically based on criteria such as burning sensation, blanching of mucosa, fibrous bands and reduced mouth opening.

## Saliva collection:

Unstimulated whole saliva was collected from all participants under standardized conditions. Participants were instructed to refrain from eating, drinking, smoking, or performing oral hygiene procedures for at least 1-2 hours prior to sample collection. Saliva was collected between 9:00 AM and 11:00 AM to minimize diurnal variation. Subjects were asked to sit comfortably and allow saliva to accumulate in the floor of the mouth and then expectorate into sterile containers. Approximately 2-5 mL of saliva was collected from each participant.

## Sample processing and storage:

The collected saliva samples were immediately transported to the laboratory and centrifuged at 3000 rpm for 10 minutes to remove debris and cellular components. The supernatant was separated and stored at -20°C until biochemical analysis.

## Biochemical analysis:

The salivary samples were analyzed for oxidative stress markers and antioxidant parameters, including:

[1] Malondialdehyde (MDA): Measured as an indicator of lipid peroxidation using the thiobarbituric acid reactive substances (TBARS) method

[2] Superoxide Dismutase (SOD): Assessed using standard enzymatic assay methods

[3] Catalase: Estimated based on its ability to decompose hydrogen peroxide

[4] Total Antioxidant Capacity (TAC): Evaluated using established spectrophotometric methods

All assays were performed according to standardized protocols using commercially available kits and readings were obtained using a spectrophotometer.

## Statistical analysis:

The collected data were entered into Microsoft Excel and analyzed using Statistical Package for the Social Sciences (SPSS) software (version 28). Descriptive statistics, including mean and standard deviation, were calculated for all variables.

[1] Comparison between groups: One-way analysis of variance (ANOVA) was used to compare the mean values of oxidative stress markers among the three groups.

[2] Post-hoc analysis: Tukey's test was applied for intergroup comparisons.

[3] Correlation analysis: Pearson's correlation coefficient was used to assess the relationship between oxidative stress markers and clinical parameters. 

[4] A p-value of less than 0.05 was considered statistically significant.

## Results:

A total of 100 subjects were included in the study and categorized into three groups: oral leukoplakia (n=35), oral submucous fibrosis (OSMF) (n=35) and healthy controls (n=30). The results were analyzed and compared for salivary oxidative stress markers, including malondialdehyde (MDA), superoxide dismutase (SOD), catalase and total antioxidant capacity (TAC). The majority of participants were males, with the highest proportion observed in the OSMF group. The mean age was higher in the leukoplakia group compared to OSMF and controls (Table 1). A statistically significant increase in MDA levels and a decrease in antioxidant markers (SOD, catalase and TAC) were observed in both leukoplakia and OSMF groups compared to controls (p < 0.001). OSMF patients showed the highest oxidative stress levels (Table 2). Post-hoc analysis revealed statistically significant differences between most groups. OSMF showed significantly higher MDA and lower antioxidant levels compared to leukoplakia and controls (Table 3). A significant positive correlation was observed between MDA levels and duration/frequency of deleterious habits, whereas antioxidant markers showed a negative correlation. In OSMF patients, reduced mouth opening was significantly associated with higher oxidative stress (Table 4). One-way ANOVA demonstrated a highly significant difference in all oxidative stress markers among the three groups (p < 0.001), indicating strong statistical evidence of altered oxidative status in oral leukoplakia and OSMF patients (Table 5).

## Discussion:

The present study was undertaken to comparatively evaluate salivary oxidative stress markers in oral leukoplakia and oral submucous fibrosis (OSMF) and to assess their potential role in disease pathogenesis. The findings of this study demonstrated a significant increase in salivary malondialdehyde (MDA) levels along with a marked decrease in antioxidant parameters such as superoxide dismutase (SOD), catalase and total antioxidant capacity (TAC) in both oral leukoplakia and OSMF groups compared to healthy controls. Furthermore, OSMF patients exhibited comparatively higher oxidative stress and lower antioxidant levels than leukoplakia patients, indicating a more severe redox imbalance. These findings strongly support the role of oxidative stress in the pathogenesis of oral potentially malignant disorders. The increased MDA levels observed in the present study indicate enhanced lipid peroxidation due to excessive generation of reactive oxygen species (ROS), while the reduction in antioxidant enzymes suggests depletion of the body's defense mechanism. Our findings are in accordance with the study conducted by Wiśniewski *et al.* (2026), [11] which reported significantly elevated salivary MDA levels and reduced antioxidant markers in patients with oral leukoplakia compared to healthy individuals. Their meta-analysis concluded that oral leukoplakia is associated with a pro-oxidant shift in salivary redox balance, supporting the concept that oxidative stress plays a crucial role in malignant transformation. Similarly, the results of the present study are consistent with the findings of Waikhom *et al.* (2026), [12] who evaluated salivary MDA levels in tobacco users with and without OSMF. They reported significantly increased MDA levels in OSMF patients compared to controls, along with a positive correlation between oxidative stress and disease severity. This aligns with our observation that OSMF patients demonstrated the highest oxidative stress levels among all groups. In agreement with our results, Ganta *et al.* (2021) [13] (salivary MDA study in OSMF) demonstrated that salivary MDA levels were significantly elevated in OSMF patients and showed a progressive increase with advancing clinical stages. This supports our correlation findings, where oxidative stress markers were associated with habit duration and severity of disease. Furthermore, Surboyo *et al.* (2024) [14] reported significant alterations in salivary oxidative stress biomarkers in OSMF patients, including increased levels of lipid peroxidation markers and decreased antioxidant status. The authors emphasized that oxidative stress plays a central role in fibrosis and disease progression, which is in line with the higher oxidative burden observed in OSMF patients in the present study. Although focusing on a different OPMD, the study by Singh *et al.* (2022) [15] on oral lichen planus also demonstrated elevated salivary MDA levels, reinforcing the concept that oxidative stress is a common underlying mechanism in various oral potentially malignant disorders. This broader evidence further strengthens the validity of our findings and highlights the significance of oxidative stress markers in oral pathology. In the present study, the decreased levels of SOD, catalase and TAC in both leukoplakia and OSMF groups indicate impaired antioxidant defense mechanisms. This reduction may be attributed to chronic exposure to tobacco and areca nut, leading to excessive ROS production that overwhelms endogenous antioxidant systems. Similar findings have been reported in previous studies by Ramesh *et al.* (2025) [16] suggesting that prolonged oxidative stress leads to enzymatic inactivation and depletion of antioxidants. Another important observation in this study was the significant correlation between oxidative stress markers and clinical parameters such as duration and frequency of deleterious habits. This suggests that the cumulative exposure to carcinogenic substances contributes to increased oxidative damage and disease progression. In OSMF patients, reduced mouth opening showed a strong association with higher MDA levels, indicating that oxidative stress may play a role in the severity of fibrosis which is consistent with the findings of Ahmed *et al.* (2025) [17]. The comparative aspect of this study provides additional insight into the differential pathogenesis of oral leukoplakia and OSMF. While both conditions showed increased oxidative stress, OSMF demonstrated a more pronounced imbalance. This may be due to the fibrotic nature of the disease and the role of areca nut alkaloids in generating ROS and promoting collagen cross-linking. In contrast, leukoplakia primarily involves epithelial dysplasia, where oxidative stress contributes to cellular atypia and malignant transformation. Saliva as a diagnostic medium proved to be highly effective in this study. Its non-invasive nature, ease of collection and direct contact with oral lesions make it an ideal biofluid for evaluating oxidative stress markers. The findings of this study reinforce the potential of salivary biomarkers as reliable tools for early detection, disease monitoring and risk assessment in oral potentially malignant disorders. However, variations in methodology, sample size and biomarker selection across different studies may account for minor differences in findings. Despite these limitations, the overall consistency in demonstrating increased oxidative stress across studies highlights its pivotal role in oral disease pathogenesis. In summary, the results of the present study are in strong agreement with previous literature, confirming that oxidative stress plays a significant role in the development and progression of oral leukoplakia and OSMF. The higher oxidative burden observed in OSMF suggests a greater risk of disease severity and potential malignant transformation. These findings underscore the importance of incorporating salivary oxidative stress markers into routine diagnostic and prognostic evaluation of oral potentially malignant disorders [18].

## Limitations:

The present study has certain limitations that should be considered while interpreting the results. Being a cross-sectional study, it does not establish a causal relationship between oxidative stress and the progression of oral leukoplakia and oral submucous fibrosis. The sample size, although adequate, was relatively limited and confined to a single institution, which may affect the generalizability of the findings to a larger population. Variations in individual habits such as type, duration and frequency of tobacco or areca nut use were self-reported and may introduce recall bias. Additionally, only selected salivary oxidative stress markers were evaluated, whereas inclusion of a broader panel of biomarkers could have provided a more comprehensive understanding of the redox status. The study also did not include longitudinal follow-up to assess changes in oxidative stress markers over time or their role in malignant transformation. Furthermore, potential confounding factors such as dietary antioxidant intake and environmental influences were not controlled, which might have influenced the salivary antioxidant levels.

## Conclusion:

We show a significant increase in salivary oxidative stress and a concomitant decrease in antioxidant defense in patients with oral leukoplakia and oral submucous fibrosis compared to healthy controls. OSMF patients exhibited a greater imbalance in oxidative status than leukoplakia, indicating a higher severity of oxidative damage. A strong association was observed between oxidative stress markers and habit-related as well as clinical parameters. Salivary biomarkers such as MDA, SOD, catalase and TAC proved to be reliable, non-invasive indicators of disease status. Thus, we show the potential role of salivary oxidative stress markers in early diagnosis, monitoring and risk assessment of oral potentially malignant disorders.

## Figures and Tables

**Table 1:** Distribution of study participants according to age and gender

**Variable**	**Leukoplakia (n=35)**	**OSMF (n=35)**	**Control (n=30)**	**Total (n=100)**
Mean Age (years)	42.6 ±8.4	38.9 ±7.6	35.2 ±6.9	-
Male (n, %)	26 (74.3%)	28 (80%)	18 (60%)	72
Female (n, %)	9 (25.7%)	7 (20%)	12 (40%)	28

**Table 2:** Comparison of salivary oxidative stress markers among groups (Mean ± SD)

**Parameter**	**Leukoplakia(n=35)**	**OSMF(n=35)**	**Control(n=30)**	**p-value**
MDA(nmol/mL)	5.82±1.12	6.45±1.28	2.96±0.84	<0.001*
SOD(U/mL)	2.14±0.52	1.88±0.47	3.21±0.63	<0.001*
Catalase(U/mL)	18.6±3.9	16.9±3.5	25.4±4.2	<0.001*
TAC(mmol/L)	0.89±0.21	0.76±0.18	1.42±0.27	<0.001*

**Table 3:** Intergroup comparison using post-hoc turkey test

**Parameter**	**Group Comparison**	**Mean Difference**	**p-value**
MDA	Leukoplakia vs OSMF	-0.63	0.041*
	Leukoplakia vs Control	2.86	<0.001*
	OSMF vs Control	3.49	<0.001*
SOD	Leukoplakia vs OSMF	0.26	0.048*
	Leukoplakia vs Control	-1.07	<0.001*
	OSMF vs Control	-1.33	<0.001*
Catalase	Leukoplakia vs OSMF	1.7	0.062
	Leukoplakia vs Control	-6.8	<0.001*
	OSMF vs Control	-8.5	<0.001*
TAC	Leukoplakia vs OSMF	0.13	0.039*
	Leukoplakia vs Control	-0.53	<0.001*
	OSMF vs Control	-0.66	<0.001*

**Table 4:** Correlation of oxidative stress markers with clinical parameters

**Parameter**	**MDA**	**SOD**	**Catalase**	**TAC**
Duration of Habit	+0.62*	-0.48*	-0.44*	-0.51*
Frequency of Habit	+0.58*	-0.42*	-0.39*	-0.46*
Mouth Opening (OSMF)	-0.55*	+0.37*	+0.33*	+0.41*
(*p < 0.05)

**Table 5:** SPSS (ANOVA) Output for comparison of oxidative stress markers

**Parameter**	**Source of Variation**	**Sum of Squares**	**df**	**Mean Square**	**F-value**	**p-value**
MDA	Between Groups	152.84	2	76.42	58.73	<0.001*
	Within Groups	126.25	97	1.3	-	-
SOD	Between Groups	42.67	2	21.33	49.12	<0.001*
	Within Groups	42.11	97	0.43	-	-
Catalase	Between Groups	812.54	2	406.27	62.88	<0.001*
	Within Groups	626.19	97	6.45	-	-
TAC	Between Groups	9.82	2	4.91	71.34	<0.001*
	Within Groups	6.67	97	0.069	-	-
